# The risk of renal comorbidities in celiac disease patients depends on the phenotype of celiac disease

**DOI:** 10.1111/joim.13532

**Published:** 2022-06-22

**Authors:** Rakel Nurmi, Camilla Pasternack, Teea Salmi, Kaisa Hervonen, Inka Koskinen, Jutta Järvelin, Heini Huhtala, Pekka Collin, Jukka Mustonen, Katri Kaukinen, Satu Mäkelä

**Affiliations:** ^1^ Celiac Disease Research Center Faculty of Medicine and Health Technology Tampere University Tampere Finland; ^2^ Department of Dermatology Tampere University Hospital Tampere Finland; ^3^ Department of Internal Medicine Central Finland Health Care District Jyväskylä Finland; ^4^ Department of Knowledge Brokers Finnish Institute for Health and Welfare Helsinki Finland; ^5^ Faculty of Social Sciences Tampere University Tampere Finland; ^6^ Department of Gastroenterology and Alimentary Tract Surgery Tampere University Hospital Tampere Finland; ^7^ Faculty of Medicine and Health Technology Tampere University Tampere Finland; ^8^ Department of Internal Medicine Tampere University Hospital Tampere Finland

**Keywords:** celiac disease, dermatitis herpetiformis, end‐stage renal disease, glomerulonephritis, IgA nephropathy, kidney disease

## Abstract

**Background:**

An increased risk of kidney disease in patients with celiac disease has been reported, but the association has remained obscure. Only few studies have investigated the association between renal comorbidities and dermatitis herpetiformis, a cutaneous manifestation of celiac disease.

**Objectives:**

We investigated whether patients with different phenotypes of celiac disease are at higher risk of kidney diseases than age‐ and sex‐matched references.

**Methods:**

The diagnoses of glomerulonephritis, diabetic nephropathy, interstitial nephritis, and end‐stage renal disease obtained from the National Hospital Discharge Register between 1970 and 2015 were identified in celiac disease (Marsh III, *n* = 1072) and dermatitis herpetiformis (*n* = 368) patients diagnosed at Tampere University Hospital catchment region and in 4296 reference subjects. Using the Cox proportional hazards model, we compared the risk of kidney diseases between patients and references. The study protocol was approved by the Regional Ethics Committee of Tampere University Hospital (R16090). As the study was register based, no consent from patients was required.

**Results:**

Even after adjusting for type 1 diabetes, celiac disease was associated with an elevated risk of kidney disease (hazard ratio [HR] 1.85, 95% confidence interval [CI] 1.12–3.03), glomerulonephritis (HR 3.37, 95% CI 1.64–6.95), and IgA nephropathy (IgAN) (HR 18.98, 95% CI 2.29–157.63). No similarly elevated risk was found among dermatitis herpetiformis patients (HR 1.50, 95% CI 0.63–3.55; HR 2.21, 95% CI 0.77–6.38; and HR 5.87, 95% CI 0.53–64.79, respectively).

**Conclusion:**

Celiac disease patients were at increased risk of kidney diseases, notably IgAN. The risk was dependent on the celiac disease phenotype and was not seen in patients with dermatitis herpetiformis. Awareness of possible renal manifestations is recommended when treating celiac disease patients.

## Graphical abstract

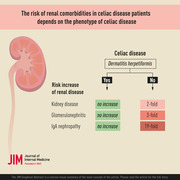

## Introduction

Malabsorption and gastrointestinal symptoms are relatively well‐known manifestations in celiac disease, which is a chronic autoimmune enteropathy. Celiac disease may also present with a variety of extraintestinal manifestations, affecting skin, the liver, or joints [1]. Dermatitis herpetiformis with a blistering rash is a well‐defined cutaneous manifestation of celiac disease [2]. Dermatitis herpetiformis—like other celiac disease phenotypes—is involved in celiac‐type enteropathy and circulating celiac‐specific autoantibodies, and an association with other autoimmune diseases has been reported [2, 3, 4]. Females predominate in celiac disease but not in dermatitis herpetiformis, and in contrast to celiac disease, dermatitis herpetiformis is rarely diagnosed in children [3, 4].

A link between celiac disease and kidney diseases, such as glomerulonephritis and diabetic nephropathy, has been recognized. However, the results are still somewhat inconsistent, especially concerning the relation between celiac disease and IgA nephropathy (IgAN) [5, 6, 7, 8, 9]. Nationwide epidemiological studies from Sweden have shown that celiac disease may be associated with end‐stage renal disease (ESRD) [8, 10], but whether these results can be generalized globally remains uncertain [10]. Moreover, the only case reports so far have shown a link between dermatitis herpetiformis and glomerulonephritis [11, 12, 13], while data concerning the risk of renal disorders in dermatitis herpetiformis are lacking.

Whether the connection between celiac disease and kidney disorders is independent or linked to associated disorders, such as type 1 diabetes, remains little known. Swedish studies have investigated the role of type 1 diabetes in an association between celiac disease and renal disorders. Welander et al. [10] showed that adjusting for type 1 diabetes changed the increase in risk for ESRD only marginally in patients with celiac disease, while another study found no overall excess risk of chronic kidney disease in individuals suffering from type 1 diabetes and celiac disease [14]. Other explanations, such as shared etiopathogenetic factors, may also account for the association between celiac disease and kidney disorders [5, 15–17]. As in celiac disease, small bowel inflammation and increased permeability have been detected in IgAN [18, 19, 20]. Celiac disease is triggered by ingested gluten and treated with a gluten‐free diet (GFD) [20]. Intriguingly, gluten may be a factor in the link between the gut and kidney, as a beneficial effect of GFD in the course of IgAN has been shown in some case reports and mice models [21, 22].

The aim of this study was to investigate with a well‐defined patient cohort and long follow‐up whether patients with celiac disease or dermatitis herpetiformis were at increased risk of renal involvement including glomerulonephritis, diabetic nephropathy, interstitial nephritis, or ESRD compared to reference individuals.

## Materials and methods

### Study patients and references

The long‐term data on the study population were obtained by enrolling all 1072 patients diagnosed with celiac disease and 368 subjects with dermatitis herpetiformis between the years 1969 and 2000 in the catchment area of Tampere University Hospital (TAUH). Celiac disease patients presented with manifestations such as gastrointestinal symptoms, malabsorption, arthralgia, and neurological problems, and some were found by screening in risk groups. Dermatitis herpetiformis patients had typical cutaneous blistering rash. Thirty patients with data irregularities concerning the unclear classification of the celiac disease phenotypes were excluded.

Diagnosis of celiac disease and dermatitis herpetiformis was based on histological findings—diagnostic small bowel mucosal damage in celiac disease (Marsh classification) and dermal IgA deposits in direct immunofluorescence in dermatitis herpetiformis [23]. The date of duodenal or skin biopsy was set as the date of diagnosis of celiac disease or dermatitis herpetiformis (index date). Patients diagnosed clinically with dermatitis herpetiformis before immunofluorescence examinations became available in the 1980s had their diagnosis biopsy confirmed later, and the date of diagnosis was taken to be the date of the original clinical diagnosis.

After being diagnosed with celiac disease and dermatitis herpetiformis, all patients were advised to adhere to a strict GFD. In addition, dapsone medication—an anti‐inflammatory drug that relieves the skin symptoms but has no effect on the small bowel mucosal damage [3]—was initiated for dermatitis herpetiformis patients with severe skin symptoms. Dermatitis herpetiformis patients were followed up at the Department of Dermatology in TAUH for at least 1–2 years or until the end of the possible treatment with dapsone. Follow‐up of celiac disease patients was organized either in primary or tertiary care, depending on the severity of the disease.

For each celiac disease and dermatitis herpetiformis patient, three reference individuals matched for age, sex, calendar period, and county were identified from the Digital and Population Data Services Agency. The date of celiac disease or dermatitis herpetiformis diagnosis was used as the index date for matching. Reference individuals (*n* = 24) with celiac disease or dermatitis herpetiformis were excluded before study entry.

### Study design

Data on the patients’ celiac disease or dermatitis herpetiformis diagnosis, such as the year of diagnosis and disease phenotype, were collected from patient records. Diagnostic codes for celiac disease and dermatitis herpetiformis in reference individuals were obtained from the Finnish National Hospital Discharge Register (NHDR). NHDR is maintained by the Finnish Institute for Health and Welfare and contains data on hospital inpatient and outpatient care, such as hospital admission and discharge days, diagnoses recorded according to the Finnish version of the International Classification of Diseases (ICD) 8–10 coding (ICD‐8 during the period 1969–1986, ICD‐9 during the period 1987–1995, and ICD‐10 since 1996), and data on the procedures implemented during hospital stay. The diagnostic codes recorded for celiac disease and dermatitis herpetiformis in reference individuals were 269.00/269.10/269.98, 5790A, K90.0 and 693.99, 6940A, L13.0, respectively.

Data on kidney comorbidities for the time period 1970–2015 for patients and references were obtained from NHDR. We collected data on diagnoses of glomerulonephritis, diabetic nephropathy, interstitial nephritis, and ESRD. ESRD was defined as having a diagnosis or procedure code of chronic dialysis or kidney transplant. For relevant ICD and procedure codes showing these renal disorders, see Table S1. The term chronic dialysis was defined as having had two or more episodes of dialysis treatment or duration of dialysis therapy exceeding 1 month before death. The specific diagnosis of IgAN was only set in the most recent version of ICD coding. Any nonacute glomerulonephritis diagnosis was taken to be chronic glomerulonephritis. It is noteworthy that some of the diagnoses in the era of the ICD‐8 classification system were not based on kidney biopsies, so the term “nephritis” was considered to represent glomerulonephritis.

Data on diabetic nephropathy were identified as described in Table S1. A distinction between diabetes types 1 and 2 was made based on ICD‐10 codes (E10 and E11, respectively) and ICD‐9 codes (250B and 250A, respectively). Of note, in the Finnish version of the ICD, the letters A and B were used to differentiate the types of diabetes. However, no such distinction could be made in the era of ICD‐8. Nevertheless, in ICD‐8, we defined diabetes mellitus type 1 as a diagnosis of diabetes before the age of 30 years. If in NHDR a patient had diagnoses of both types of diabetes, the classification was made according to the type of diabetes appearing more often in the patient's diagnosis codes. If there was a specific diagnosis of either type (1 or 2) of diabetic nephropathy, this determined the type of diabetes mellitus. Such diagnosis of diabetic nephropathy established by a professional in internal medicine was considered most reliable.

If the initial diagnosis of kidney disorder was unspecific, a more precise later diagnosis was used to describe the first renal comorbidity identified if considered from a nephrological perspective to be the same disorder. If two or more distinct kidney diseases were found, these were registered separately.

Follow‐up began on 1 January 1970 or birth thereafter and ended on the date of the first diagnosis of renal comorbidity, death, emigration, or the end of the study period (31 December 2015), whichever occurred first. Therefore, in most patients, the follow‐up began before the celiac disease or dermatitis herpetiformis diagnosis. Data on death and emigration were obtained from the Population Register Centre of Finland.

### Statistical analysis

Quantitative data were expressed as medians and ranges, and categorized values as numbers and percentages. The χ2‐test was used to assess differences between categorized variables. Incidence rates for 10,000 person‐years (p‐y) with 95% confidence intervals (CIs) for the studied kidney diseases were calculated, and the Cox proportional hazards model was used to compare the risk of renal comorbidities between patients and matched references. When calculating the risk of renal comorbidities separately for celiac disease and dermatitis herpetiformis, patients were compared to their own matched reference groups. Type 1 diabetes recorded from NHDR during the entire follow‐up time was used for adjustment. A *p*‐value < 0.05 was considered statistically significant. All statistical analyses were performed with SPSS version 26 (IBM Corporation, Armonk, NY, USA) or Stata version 16 (StataCorp, College Station, Texas, USA) in co‐operation with a statistician.

## Results

Of 1072 patients with celiac disease, 68% were female, whereas among the 368 patients with dermatitis herpetiformis the gender distribution was equal (Table 1). Median age at the time of celiac disease or dermatitis herpetiformis diagnosis was 39 years in both patient groups. All celiac disease patients and 71% of the subjects with dermatitis herpetiformis had Marsh III‐type small bowel mucosal lesions at the time of diagnosis. Celiac disease and dermatitis herpetiformis patients and reference individuals were all followed up for a median of 46 years. Type 1 diabetes occurred more often in patients with celiac disease than in patients with dermatitis herpetiformis (6.2% vs. 1.9%, *p* = 0.001) (Table 1).

**Table 1:** Demographic data on 1072 patients with celiac disease, 368 subjects with dermatitis herpetiformis, and 4296 age‐ and sex‐matched reference individuals

	Celiac disease *n* = 1072		Dermatitis herpetiformis *n* = 368		Reference individuals *n* = 4296	
	*n*/median	%/range	*n*/median	%/range	*n*/median	%/range
Age (at index date^a^), years	39	1–85	39	5–84	39	1–85
Age (at the end of follow‐up), years	61	9–97	68	23–96	63	6–101
Index year^b^	1990	1966–2001	1984	1970–2000	1989	1966–2001
Female	725	67.6	180	48.9	2695	62.7
Whole follow‐up, years	46	1–46	46	7–46	46	5–46
DM	115	10.7^c^	29	7.9^d^	380	8.8
DM1	66	6.2^c^	7	1.9	44	1.0
DM2	49	4.6^d^	22	6.0^d^	336	7.8
Hypertension	186	17.4	69	18.8	747	17.4

Abbreviations: DM, diabetes mellitus; DM1, type 1 diabetes; DM2, type 2 diabetes.

^a^ Index date is the date of diagnosis of celiac disease or dermatitis herpetiformis.

^b^ Index year is the year of the diagnosis of celiac disease or dermatitis herpetiformis.

^c^ Statistically significantly increased compared to matched references.

^d^ Statistically significantly decreased compared to matched references.

As shown in Table 2, celiac disease was positively associated with the presence of kidney disease, even after adjusting for type 1 diabetes (*n* = 37, incidence = 8.40/10,000 p‐y, adjusted hazard ratio [HR] 1.85, 95% CI 1.12–3.03). The adjusted risk of having any glomerulonephritis was threefold in patients with celiac disease compared to that of matched individuals (*n* = 17, incidence = 3.84/10,000 p‐y, 95% CI 1.64–6.95). The elevated risk of IgAN was over 18‐fold among celiac disease patients compared to that of individuals in the reference group, and the increased risk remained statistically significant when adjusting for type 1 diabetes (*n* = 6, incidence = 1.35/10,000 p‐y, 95% CI 2.29–157.63) (Table 2). Increased risk of type 1 diabetic nephropathy was over fivefold among celiac disease patients compared to that of reference individuals (95% CI 2.20–11.50). Interstitial nephritis was associated with celiac disease also after adjusting for type 1 diabetes (adjusted HR 4.49, 95% CI 1.04–19.43) (Table 2).

**Table 2:** Incidence and risk of renal comorbidities in celiac disease patients and in age‐ and sex‐matched reference individuals in long‐term follow‐up

	Patients with celiac disease, *n* = 1072			Reference individuals, *n* = 3197						
	*n*	Incidence/10,000 p‐y	95% CI	*n*	Incidence/10,000 p‐y	95% CI	HR	95% CI	Adjusted^a^ HR	95% CI
Kidney disease^b^	37	8.40	6.09–11.60	36	2.70	1.95–3.75	3.13	1.98–4.95	1.85	1.12–3.03
GN	17	3.84	2.39–6.18	14	1.05	0.62–1.77	3.67	1.81–7.45	3.37	1.64–6.95
Acute GN	3	0.67	0.22–2.09	2	0.15	0.04–0.60	4.52	0.76–27.04	3.61	0.56–23.12
Chronic GN	14	3.16	1.87–5.33	12	0.90	0.51–1.58	3.52	1.63–7.62	3.33	1.52–7.29
IgAN	6	1.35	0.61–3.01	1	0.07	0.01–0.53	18.05	2.17–149.90	18.98	2.29–157.63
Other than IgAN	8	1.80	0.90–3.60	11	0.82	0.46–1.49	2.19	0.88–5.45	1.93	0.75–4.95
Diabetic nephropathy, DM1	15	3.38	2.04–5.61	9	0.67	0.35–1.30	5.03	2.20–11.50	–	–
Diabetic nephropathy, DM2	4	0.90	0.34–2.39	10	0.75	0.40–1.39	1.21	0.38–3.85	–	–
Interstitial nephritis	5	1.12	0.47–2.70	3	0.22	0.07–0.70	5.02	1.20–20.99	4.49	1.04–19.43
End‐stage renal disease	13	2.93	1.70–5.04	7	0.52	0.25–1.10	5.62	2.24–14.08	2.28	0.84–6.21

Abbreviations: CI, confidence interval; DM1, type 1 diabetes; DM2, type 2 diabetes; GN, glomerulonephritis; HR, hazard ratio; IgAN, IgA nephropathy; p‐y, person‐years.

^a^ Adjusted for DM1.

^b^ Kidney disease includes any glomerulonephritis, diabetic nephropathy, and interstitial nephritis.

We observed over fivefold elevated risk of ESRD in celiac disease patients (95% CI 2.24–14.08), but type 1 diabetes had a significant role in the association (adjusted HR 2.28, 95% CI 0.84–6.21) (Table 2). Table 3 shows the characteristics of celiac disease patients and age‐ and sex‐matched reference individuals with ESRD. Nine (69%) out of 13 celiac disease patients had type 1 diabetic nephropathy as a cause of ESRD, while the same etiological diagnosis behind ESRD was found in only three (43%) out of seven reference individuals. Median age at the time of ESRD diagnosis was 46 and 60 years in the celiac disease patients and the reference individuals, respectively.

**Table 3:** Characteristics of celiac disease patients and age‐ and sex‐matched reference individuals with end‐stage renal disease

	CD patients, *n* = 13	References, *n* = 7
Female, *n* (%)	9 (69)	5 (71)
Underlying kidney disease, *n* (%)^a^		
DM1 nephropathy	9 (69)	3 (43)
DM2 nephropathy	1 (8)	0 (0)
Any glomerulonephritis	4 (31)	2 (29)
Interstitial nephritis	2 (15)	0 (0)
Not known	0 (0)	2 (29)
DM, *n* (%)	10 (77)	4 (57)
DM1, *n* (%)	9 (69)	3 (43)
DM2, *n* (%)	1 (8)	1 (14)

Abbreviations: CD, celiac disease; DM, diabetes mellitus; DM1, type 1 diabetes; DM2, type 2 diabetes.

^a^ Two different renal diagnoses were observed in three individuals with celiac disease.

When the risk of renal comorbidities was assessed separately for dermatitis herpetiformis patients, no increase in the risk of kidney diseases was seen (*n* = 8, incidence = 5.17/10,000 p‐y, adjusted HR 1.50, 95% CI 0.63–3.55). No differences in the risks of glomerulonephritis (*n* = 6, incidence = 3.87/10,000 p‐y, adjusted HR 2.21, 95% CI 0.77–6.38) or IgAN (*n* = 2, incidence = 1.28/10,000 p‐y, adjusted HR 5.87, 95% CI 0.53–64.79) could be shown between dermatitis herpetiformis patients and references (Table 4). More specifically, no statistically significantly elevated risks were found in any of the renal outcomes studied, and no cases with ESRD were observed among patients with dermatitis herpetiformis.

**Table 4:** Incidence and risk of renal comorbidities in patients with dermatitis herpetiformis and in age‐ and sex‐matched reference individuals in long‐term follow‐up

	Patients with DH, *n* = 368			Reference individuals, *n* = 1099						
	*n*	Incidence/10,000 p‐y	95% CI	*n*	Incidence/10,000 p‐y	95% CI	HR	95% CI	Adjusted^a^ HR	95% CI
Kidney disease^b^	8	5.17	2.58–10.33	15	3.33	2.01–5.53	1.54	0.65–3.62	1.50	0.63–3.55
GN	6	3.87	1.74–8.62	8	1.78	0.89–3.55	2.19	0.76–6.31	2.21	0.77–6.38
Acute GN	2	1.28	0.32–5.12	0	0	–	–	–	–	–
Chronic GN	4	2.57	0.96–6.85	7	1.55	0.74–3.26	1.65	0.48–5.65	1.67	0.49–5.71
IgAN	2	1.28	0.32–5.12	1	0.22	0.03–1.57	5.82	0.53–64.14	5.87	0.53–64.79
Other than IgAN	2	1.28	0.32–5.13	6	1.33	0.60–2.96	0.96	0.19–4.76	0.97	0.20–4.81
Diabetic nephropathy, DM1	0	0	–	1	0.22	0.03–1.57	–	–	–	–
Diabetic nephropathy, DM2	1	0.64	0.09–4.53	4	0.88	0.33–2.36	0.69	0.08–6.17	–	–
Interstitial nephritis	1	0.64	0.09–4.53	2	0.44	0.11–1.77	1.40	0.13–15.43	1.41	0.13–15.58
End‐stage renal disease	0	0	–	3	0.66	0.21–2.06	–	–	–	–

Abbreviations: CI, confidence interval; DH, dermatitis herpetiformis; DM1, type 1 diabetes; DM2, type 2 diabetes; GN, glomerulonephritis; HR, hazard ratio; IgAN, IgA nephropathy; p‐y, person‐years.

^a^ Adjusted for DM1.

^b^ Kidney disease includes any glomerulonephritis, diabetic nephropathy, and interstitial nephritis.

## Discussion

This comprehensive register‐based study aiming to investigate the risk of renal comorbidities in celiac disease and dermatitis herpetiformis patients identified an increased risk for kidney diseases and especially glomerulonephritis among celiac disease patients compared to matched reference individuals. Patients with dermatitis herpetiformis did not show increased risk for kidney diseases.

The present finding of an elevated risk of renal manifestations among celiac disease patients corroborates earlier reports [8, 10, 24]. The most obvious association was found between celiac disease and IgAN, as celiac disease patients had a 19‐fold increased risk of concomitant IgAN during follow‐up. The earlier studies have reported mainly parallel results [5, 24]. Even though the association has been recognized, the absolute risk of renal disease in celiac disease has been fairly low [10, 24]. According to the present study, the difference in incidence rates for kidney disease was ca. 5.7/10,000 p‐y, and accordingly, six extra cases of renal disease could be seen among 1000 celiac disease patients in 10 years, which actually suggests a fairly low absolute risk. The concept of a gut–renal axis—especially the link between celiac disease and IgAN—has attracted increased attention in the study field, and possible common factors—such as IgA responses, the function of tissue transglutaminase, and reactivity to gluten—have been suggested to explain this association [15, 16, 25]. Despite these potential links, as far as we know, no routine screening for kidney diseases has been recommended for celiac disease patients, nor has screening for celiac disease in IgAN patients been routinely suggested.

The rising prevalence of celiac disease has been recognized [1], and chronic kidney disease has likewise become a growing global health issue, sometimes preluding the development of ESRD [26, 27]. Diabetes and glomerulonephritis are common causes of chronic kidney disease [26, 28]. In clinical practice, it would be important to remember that celiac disease patients may suffer from kidney disorders and especially be at increased risk of IgAN. In this way, the possible renal comorbidities could be recognized as early as possible in order to prevent the progression of kidney diseases in celiac disease patients [29]. In the present study, the risk of ESRD in celiac disease patients was mainly associated with type 1 diabetes; thus, the link between celiac disease, diabetes, and nephrological disorders should be borne in mind. What is known about the role of celiac disease in the risk of nephropathy in type 1 diabetes is inconsistent, with reports of both positive associations [30, 31] and no obvious contribution of celiac disease [14, 32]. In more detail, even though showing overall no excess risk of chronic kidney disorder in patients with celiac disease and type 1 diabetes, one of the abovementioned studies established a positive link between longstanding celiac disease and chronic renal disorder in type 1 diabetes [14]. The other study investigating the risk of ESRD in celiac disease showed that the effect of type 1 diabetes on the risk was only marginal [10]. However, the review by Boonpheng et al. [29] concludes that celiac disease may be independently linked with kidney dysfunction regardless of type 1 diabetes.

It is of interest that in this study, the patients with dermatitis herpetiformis differed from the other celiac disease patients in their risk of renal disorders. To the best of our knowledge, this is a novel finding, although a nonsignificant difference in the prevalence of glomerulonephritis between patients with dermatitis herpetiformis and celiac disease (0.3% vs. 0.8%) has been described [33]. The explanations for the observed difference in the risk of kidney diseases between phenotypes are unclear. Increased intestinal permeability and mucosal inflammation have been suggested to link bowel diseases and glomerulonephritis [15], and because celiac‐type enteropathy is milder in dermatitis herpetiformis than in celiac disease [2, 3], the bowel pathology may affect the different risk of renal involvement. In celiac disease, the autoantibodies target tissue transglutaminase (TG2), whereas in dermatitis herpetiformis autoantibodies are generated against both TG2 and epidermal transglutaminase (TG3). These differences in immunological processes may affect the systemic nature of the disease phenotypes [3, 4, 34]. Moreover, a slightly increased mortality risk has been observed in patients with celiac disease, whereas the mortality rate in dermatitis herpetiformis is decreased [2, 4]. According to a Finnish cohort study [35], strict adherence to a GFD, less smoking, and less hypercholesterolemia in patients with dermatitis herpetiformis could explain the favorable prognosis of the disease, which could also reflect the decreased risk of kidney comorbidities. The use of dapsone, the anti‐inflammatory drug used in the treatment of dermatitis herpetiformis, could also have some effect on the risk of renal disorders.

The main strengths of this study were the well‐defined, biopsy‐confirmed celiac disease and dermatitis herpetiformis patients, which enabled us to analyze the risk separately for different phenotypes of the disease. In addition, the follow‐up time in this study was long. As both celiac disease and many kidney disorders can develop gradually and manifest at any age, the follow‐up covering periods both before and after celiac disease and dermatitis herpetiformis diagnosis can be regarded as a strength of the study. Further, although this was a register study, all the renal diagnoses were evaluated separately for each individual in order to achieve more precise estimates.

Some potential weaknesses should also be discussed. Due to the nature of a register study, we were not able to exclude the risk that some reference individuals might have had undiagnosed celiac disease or dermatitis herpetiformis. Furthermore, there remains the possibility that some renal diagnoses were not registered in NHDR or that some individuals had undetected renal disorders. Some issues concerning the previous ICD classifications should also be discussed. In the Finnish National Classification of Diseases, ICD‐8 diagnostic codes 580–583 referred to clinically diagnosed “nephritis.” Hence, no precise differentiation between glomerular or tubular diseases was feasible, and these diagnoses were classified as glomerulonephritis. The diagnosis of interstitial nephritis was not included in ICD‐8, and in both ICD‐8 and ICD‐9 the diagnosis of IgAN was missing. Also, we had no data on body mass index, laboratory values (including albuminuria and glomerular filtration rate), smoking status or other lifestyle factors, or on adherence to GFD. Nor was liver disease considered as a theoretically potential confounding factor, which could have been associated with celiac disease or IgAN.

## Conclusion

Our study suggests that awareness of possible associated renal diseases, especially glomerulonephritis, is necessary when treating patients with celiac disease. At least an easily accessible urine dipstick test and serum creatinine measurements could be used to exclude these renal comorbidities in celiac disease. Serologic testing for celiac disease in IgAN patients should also be considered. Further investigations are needed to understand the risk of renal disorders in the different phenotypes of celiac disease.

## Funding

This study was supported by the Competitive State Research Financing of the Expert Responsibility Area of Tampere University Hospital, the Academy of Finland, the Sigrid Jusélius Foundation, the Finnish Celiac Society, Tampere University Doctoral School, the Finnish Kidney and Liver Association, and the Emil Aaltonen Foundation.

## Conflict of interest

The authors declare no conflict of interest.

## Author contributions

Rakel Nurmi: Conceptualization; formal analysis; funding acquisition; investigation; methodology; visualization; writing – original draft; writing – review and editing. Camilla Pasternack: Conceptualization; formal analysis; funding acquisition; investigation; methodology; writing – review and editing. Teea Salmi: Conceptualization; investigation; project administration; writing – review and editing. Kaisa Hervonen: Investigation; writing – review and editing. Inka Koskinen: Investigation; writing – review and editing. Jutta Järvelin: Investigation; writing – review and editing. Heini Huhtala: Formal analysis; methodology; writing – review and editing. Pekka Collin: Investigation; writing – review and editing. Jukka Mustonen: Conceptualization; investigation; methodology; supervision; writing – review and editing. Katri Kaukinen: Conceptualization; funding acquisition; investigation; methodology; project administration; supervision; writing – review and editing. Satu Mäkelä: Conceptualization; formal analysis; investigation; methodology; project administration; supervision; writing – review and editing. All authors approved the final version of the manuscript.

## Supporting information


**Table S1**. Classification of kidney diseases according to the Finnish version of the International Classification of Diseases (ICD) codes and procedure codes during the study period 1970–2015.
