# Supplementary material for: The risk of renal comorbidities in celiac disease patients depends on the phenotype of celiac disease
Source: J Intern Med. 2022 Jun 22;292(5):779–87. doi: 10.1111/joim.13532 (PMC9796855; doi:10.1111/joim.13532)
Supplement: Supplementary file 1 — Table S1. Classification of kidney diseases according to the Finnish version of the International Classification of Diseases (ICD) codes and procedure codes during the study period 1970–2015. [file JOIM-292-779-s001.docx]

Supplementary Table 1. Classification of kidney diseases according to the Finnish version of the International Classification of Diseases (ICD) codes and procedure codes during the study period 1970-2015.

|  | ICD-10 | ICD-9 | ICD-8 |
| --- | --- | --- | --- |
| **Kidney diseases** |  |  |  |
| Glomerulonephritis | N00-N08 (excluding N08.3) | 580, 581, 582 | 580, 581, 582, 583 |
| Acute glomerulonephritis | N00, N01 | 580 | 580 |
| Chronic glomerulonephritis | N02-N08 (excluding N08.3) | 581, 582 | 581, 582 |
| IgA nephropathy | N08.2*D89.80 | - | - |
| Diabetic nephropathy | E10.2, E11.2, E12.2, E13.2, E14.2, N08.3 | 2503 | 25004 |
| Interstitial nephritis | N11.8, N11.9 | 583 | - |
| **End-stage renal disease** |  |  |  |
| Chronic dialysis^a^ | Z49.1, Z49.2, Z99.2  TK800, TK820 | 6112 | Y2901  6112 |
| Renal transplantation^a^ | Z94.0  KAS10, KAS20 | 7151 | 7151 |

^a^Until 1996 the procedure codes are classified according to the Finnish Hospital League and thereafter according to the Finnish version of Nordic Medico-Statistical Committee (NOMESCO) Classification
